# Cumulative Burden of Neonatal Morbidities and Its Impact on Medical Costs and Length of Stay in Preterm Infants: A Nationwide Study in Korea

**DOI:** 10.3390/children13060779

**Published:** 2026-06-03

**Authors:** Seung Hwan Baek, Young Mi Park, Teahyen Cha, So Jin Yoon, Jung Ho Han, Jeong Eun Shin, Ho Seon Eun, Min Soo Park, Joohee Lim, Soon Min Lee

**Affiliations:** Department of Pediatrics, Yonsei University College of Medicine, Seoul 03722, Republic of Korea; baekga@yuhs.ac (S.H.B.); ympark0907@yuhs.ac (Y.M.P.); teahyencha@yuhs.ac (T.C.); sojinyoon@yuhs.ac (S.J.Y.); feagd@yuhs.ac (J.H.H.); golden-week@yuhs.ac (J.E.S.); hseun@yuhs.ac (H.S.E.); minspark@yuhs.ac (M.S.P.)

**Keywords:** infant, premature, intensive care units, neonatal, comorbidity, length of stay (LOS), health care costs

## Abstract

**Highlights:**

**What are the main findings?**

- Major neonatal morbidities in preterm infants were associated with prolonged NICU hospitalization and increased medical costs in a nationwide Korean cohort.
- Increasing numbers of coexisting neonatal morbidities were associated with progressively longer NICU length of stay and higher healthcare utilization, with the strongest associations observed in extremely preterm infants.

**What are the implications of the main findings?**

- Prevention and early management of multiple coexisting neonatal morbidities may help reduce both clinical burden and healthcare resource utilization in preterm infants.
- Nationwide variation in NICU length of stay suggests that institutional practice patterns and discharge policies may influence healthcare utilization beyond patient-level clinical factors.

**Abstract:**

Background: Neonatal morbidities are major determinants of clinical outcomes and healthcare utilization in preterm infants. However, population-level evidence quantifying the cumulative contribution of neonatal morbidities to neonatal intensive care unit (NICU) length of stay (LOS) and medical costs remains limited. Methods: We conducted a nationwide retrospective cohort study using the Korean Health Insurance Review and Assessment Service database. Preterm infants admitted to NICUs between 2020 and 2023 were identified. After exclusions, 30,034 infants with complete birth weight data and 31,240 with complete gestational age data were included. Major neonatal morbidities—including bronchopulmonary dysplasia (BPD), patent ductus arteriosus (PDA), sepsis, intraventricular hemorrhage (IVH), retinopathy of prematurity (ROP), necrotizing enterocolitis (NEC), and periventricular leukomalacia (PVL)—were identified using ICD-10 odes. Associations of individual morbidities and cumulative morbidity burden with NICU LOS and medical costs were evaluated using multivariable regression and generalized linear mixed models. Results: Mean NICU LOS was 26.5 days, and mean total medical cost was 41.9 million KRW. All major morbidities were associated with prolonged LOS and increased costs. BPD showed the strongest association with LOS, whereas NEC and sepsis were associated with the highest costs. NICU LOS and medical costs increased in a stepwise manner with increasing numbers of morbidities; each additional morbidity was associated with an 8.0-day increase in LOS and a 32.5 million KRW increase in medical costs (both *p* < 0.001). Conclusions: Greater cumulative morbidity burden was associated with prolonged hospitalization and increased healthcare costs in preterm infants.

## 1. Introduction

Preterm birth is a leading cause of neonatal morbidity, mortality, and long-term neurodevelopmental impairment worldwide [1]. While advancements in perinatal and neonatal care have significantly improved the survival of preterm infants, this progress has been accompanied by an increasing burden of prematurity-related complications [2]. Major morbidities—including bronchopulmonary dysplasia (BPD), sepsis, necrotizing enterocolitis (NEC), retinopathy of prematurity (ROP), periventricular leukomalacia (PVL), patent ductus arteriosus (PDA) and intraventricular hemorrhage (IVH)—not only adversely affect clinical outcomes but also contribute to prolonged hospitalization and intensive healthcare utilization [3,4,5].

Length of stay (LOS) in the neonatal intensive care unit (NICU) serves as a critical indicator of clinical severity and resource consumption. Prolonged LOS of these infants in the NICU has important implications for hospital occupancy, healthcare resource utilization, overall costs, and the psychological well-being of parents [6]. Although lower gestational age and birth weight are well-established predictors of prolonged LOS [7], other factors—including geographic variation, implementation of family-integrated care, and parental education—have also been shown to influence LOS [8,9]. Furthermore, while LOS reflects clinical burden, medical costs represent the direct economic impact of neonatal care. As the determinants of these two outcomes may not perfectly align, understanding their differential drivers is essential for optimizing clinical management and healthcare resource allocation.

Despite growing clinical interest, most studies have focused on individual morbidities or single outcomes within limited institutional cohorts [10,11,12,13,14], There remains a paucity of comprehensive, population-level evaluations regarding the cumulative burden of multiple morbidities and their simultaneous effects on both LOS and medical costs. Nationwide administrative databases offer a unique opportunity to address this gap, capturing representative cohorts and multidisciplinary interventions that reflect the true socioeconomic challenges faced by families and national healthcare budgets.

In South Korea, despite various national policies supporting high-risk newborns, nationwide data quantifying the economic and clinical impact of cumulative neonatal morbidities remain limited [15,16]. A clearer understanding of the relationship between morbidity burden and its impact on LOS and total costs is crucial for developing targeted intervention strategies and informing policy decisions.

Parents of infants with complex medical conditions frequently express concerns about current and future medical expenses, and report a desire to discuss financial issues with clinicians [17,18]. However, such discussions remain limited. Understanding the drivers of NICU LOS and medical costs may therefore help clinicians better counsel families regarding both clinical outcomes and economic expectations.

This study aimed to evaluate the impact of major neonatal morbidities on NICU LOS and medical costs among preterm infants using a nationwide database from the Health Insurance Review and Assessment Service (HIRA). Specifically, we investigated: (1) the independent effects of individual morbidities and baseline characteristics, and (2) the differential patterns of influence on clinical (LOS) and economic (cost) outcomes. By analyzing the cumulative burden of these conditions, we sought to provide robust evidence on the socioeconomic implications of prematurity and to offer insights to guide neonatal care policy in the modern era.

## 2. Materials and Methods

This retrospective cohort study was conducted using the HIRA database (HIRA dataset no. M20250501014), which contains nationwide claims data covering nearly the entire population of Korea. To protect patient privacy, all identifiable variables, including claim-, individual-, and organizational-level identification numbers, were randomly regenerated by the HIRA database. Preterm infants admitted to NICUs between January 2020 and December 2023 were identified. A total of 41,338 preterm infants were included in the analysis. Patients who died within 24 h of birth, were transferred, or had missing gestational age or birth weight data were excluded. Infants were stratified according to gestational age and birth weight categories to evaluate differences in clinical characteristics and outcomes. Accordingly, 30,034 infants with complete birth weight data and 31,240 infants with complete gestational age data were included in the respective analyses. Major neonatal morbidities—including BPD, PDA, sepsis, IVH, ROP, NEC, and PVL—were identified using diagnostic codes from the HIRA database. Detailed operational definitions and ICD-10/KCD-10 codes used to identify neonatal morbidities are provided in Table A1, Table A2 and Table A3. The analysis was restricted to the initial NICU hospitalization, and both medical costs and length of stay were calculated for the period from admission to discharge during the first hospitalization. Medical costs are presented in Korean Won (KRW). For international interpretability, approximate USD equivalents were calculated using an average exchange rate of 1 USD = 1200 KRW during the study period. Medical costs were calculated using reimbursement claims recorded in the Korean Health Insurance Review and Assessment Service (HIRA) database. Under the Korean National Health Insurance system, most NICU care for very low birth weight infants is covered by insurance with minimal patient cost-sharing. Therefore, reimbursement claims were considered a reasonable proxy for actual healthcare expenditures incurred during hospitalization. The cumulative number of neonatal morbidities was calculated for each infant and used as a variable representing overall morbidity burden. Temporal trends in the incidence of major neonatal morbidities were evaluated using the Mantel–Haenszel chi-square test and the Cochran–Armitage trend test. Because NICU length of stay (LOS) and medical cost data demonstrated right-skewed distributions, to evaluate associations between major neonatal morbidities and clinical outcomes, generalized linear models (GENMOD) were used after adjustment for gestational age and birth weight. For LOS analyses, GENMODs with an identity link were applied, and estimates are presented as adjusted mean differences in hospitalization duration (days). For medical cost analyses, GENMODs with a log link were used, and estimates are presented as exponentiated regression coefficients exp(β), representing multiplicative relative increases in medical costs. An exp(β) value greater than 1 indicates increased medical costs associated with the corresponding morbidity relative to infants without that condition. Reference groups were infants without the corresponding morbidity and the specified gestational age category. In separate analyses evaluating cumulative morbidity burden, regression models were additionally used to estimate changes in NICU LOS and medical costs according to the number of coexisting morbidities. For clinical interpretability, estimates derived from log-transformed models were converted back to the original scale and presented as additional days of hospitalization and additional medical costs.

To account for inter-institutional heterogeneity and clustering across NICUs, generalized linear mixed models (GLMMs) were additionally applied with hospital of birth included as a random effect. Analyses were stratified by gestational age and birth weight groups to evaluate subgroup-specific associations and partially account for differences in baseline maturity and growth characteristics. Several clinically important covariates, including antenatal steroid exposure, infant sex, SGA status, delivery mode, and NICU-level capacity variables, were not available in the HIRA database and therefore could not be included in the adjustment models. The study protocol received approval from the Institutional Review Board (IRB) of Gangnam Severance Hospital (IRB No. 3-2025-0040). Given the retrospective study design, the requirement for informed consent was waived.

## 3. Results

### 3.1. Temporal Trends in Baseline Characteristics, NICU Length of Stay, and Medical Costs

**Figure 1:**
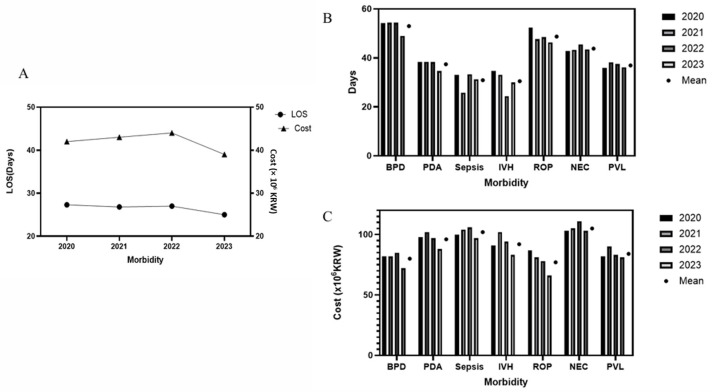
(**A**) Temporal trends in NICU length of stay across major neonatal morbidities. (**B**,**C**) Bars represent the annual NICU length of stay for each morbidity from 2020 to 2023, and points indicate the mean values across the study period. Overall NICU length of stay remained relatively stable over time at the population level. However, morbidity-specific analyses revealed notable year-to-year variability, with the magnitude of fluctuations differing across individual conditions. BPD, bronchopulmonary dysplasia; IVH, intraventricular hemorrhage; KRW, Korean won; NEC, necrotizing enterocolitis; PDA, patent ductus arteriosus; PVL, periventricular leukomalacia; ROP, retinopathy of prematurity.

A total of approximately 30,000 preterm infants were included in the analysis. The majority of infants had a birth weight ≥ 1500 g (77.1%) and gestational age ≥ 32 weeks (75.5%), whereas infants with extremely low birth weight (<500 g) and extreme prematurity (<26 weeks gestation) comprised a relatively small proportion of the cohort. Across the study period, the distributions of birth weight and gestational age remained largely stable, with only minor year-to-year fluctuations. Overall, NICU LOS showed no substantial change over time in the total population. However, when stratified by individual morbidities, considerable variability emerged across years. The magnitude of these fluctuations differed by morbidity type, indicating heterogeneous temporal patterns rather than a uniform trend (Figure 1).

The overall NICU LOS differed substantially according to the presence of major neonatal morbidities. The mean LOS in the overall population was 26.5 days, whereas infants with major morbidities showed markedly prolonged hospitalization.

Among individual conditions, BPD was associated with the longest LOS (53.1 d), followed by ROP (48.0 d), NEC (43.6 d), PDA (37.5 d), IVH (32.8 d), and sepsis (32.8 d). PVL was also associated with prolonged LOS compared to the overall population.

Across all morbidities, LOS was consistently higher in affected infants compared to the total population, demonstrating the substantial clinical burden associated with these conditions. A similar pattern was observed for medical costs. The mean total medical cost in the overall population was approximately 41.9 million KRW, whereas infants with major morbidities incurred substantially higher costs. NEC was associated with the highest medical cost (104.9 million KRW), followed by sepsis (101.9 million KRW), PDA (96.4 million KRW), IVH (92.5 million KRW), ROP (76.8 million KRW), BPD (80.5 million KRW), and PVL (84.2 million KRW).

These findings indicate that all major neonatal morbidities are associated with a considerable increase in healthcare resource utilization.

### 3.2. Morbidity Incidence and Burden Trends

The incidence of major neonatal morbidities was inversely associated with gestational age and birth weight, with the highest rates observed in the most premature and lowest birth weight infants. Across all morbidities, the presence of disease was consistently associated with increased medical costs and prolonged NICU length of stay, although the magnitude of these effects varied by condition.

BPD incidence increased with decreasing gestational age and birth weight (*p* < 0.001 for trend). Infants with BPD had substantially higher medical costs and longer NICU LOS across all strata, with the greatest differences observed in extremely preterm and low birth weight groups.

PDA incidence showed a strong inverse relationship with gestational age and birth weight (*p* < 0.001). Infants with PDA consistently incurred higher medical costs and longer NICU stays, particularly in the most premature and lowest birth weight groups.

Sepsis incidence decreased with increasing gestational age and birth weight (*p* < 0.001). The presence of sepsis was associated with higher medical costs and prolonged NICU LOS across most groups, although differences in LOS were less consistent.

IVH incidence was inversely associated with gestational age and birth weight (*p* < 0.001). Infants with IVH showed increased medical costs and modestly prolonged NICU stays, with variability across subgroups. ROP incidence decreased markedly with increasing gestational age and birth weight (*p* < 0.001). Infants with ROP had higher medical costs and substantially longer NICU LOS, with the most pronounced differences in extremely preterm infants. NEC incidence demonstrated a strong inverse trend with gestational age and birth weight (*p* < 0.001). Infants with NEC had increased medical costs and longer NICU stays, although the magnitude of differences varied across groups. PVL incidence showed a less consistent but overall decreasing trend with increasing gestational age and birth weight (*p* < 0.001). PVL was associated with higher medical costs and longer NICU stays, but with relatively smaller and more variable differences compared to other morbidities.

Overall, major neonatal morbidities were more prevalent in lower gestational age and birth weight infants and were consistently associated with increased healthcare utilization, with BPD and PDA showing the greatest impact on both medical costs and LOS.

### 3.3. Cumulative Burden of Neonatal Morbidities on Length of Stay and Medical Cost

**Figure 2:**
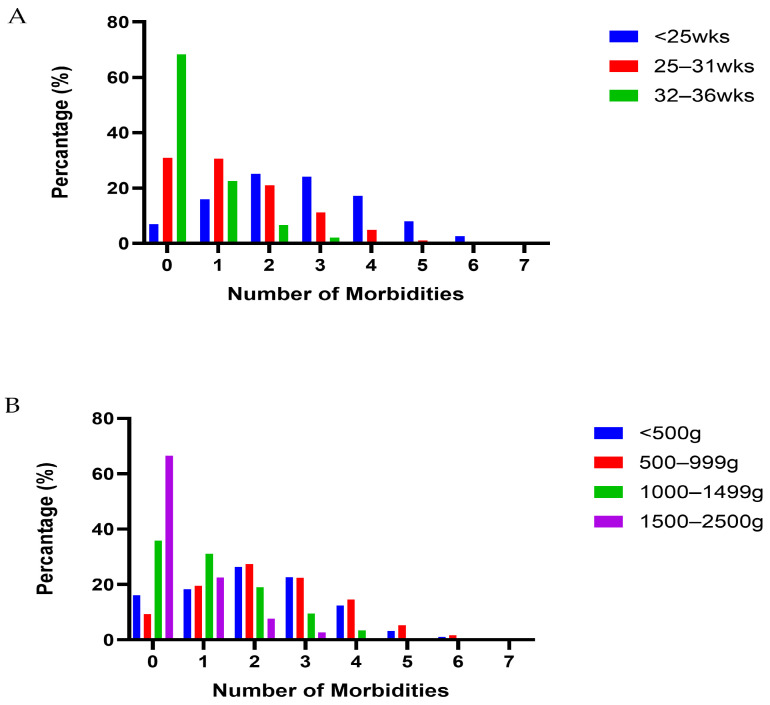
Distribution of cumulative neonatal morbidities by gestational age and birth weight. (**A**) Distribution of the number of morbidities according to gestational age groups. (**B**) Distribution of the number of morbidities according to birth weight groups. The distribution of cumulative neonatal morbidities varied by gestational age. Infants with lower gestational age showed a higher proportion of multiple morbidities, whereas those with higher gestational age were more likely to have no or fewer morbidities.

The distribution of the cumulative number of major neonatal morbidities differed according to gestational age and birth weight (Figure 2). Infants with lower gestational age and lower birth weight were more likely to have multiple coexisting morbidities, whereas those with higher gestational age and birth weight more frequently had no or fewer complications.

**Figure 3:**
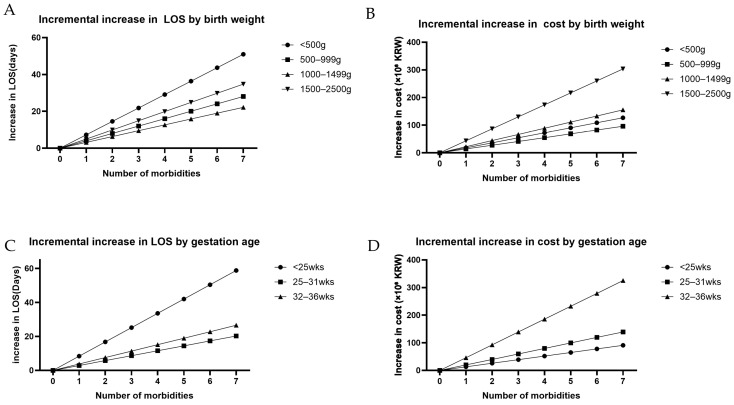
Incremental impact of cumulative neonatal morbidities on NICU length of stay and medical costs across gestational age groups and birth weight groups (**A**,**C**) Increase in NICU length of stay (LOS) according to the number of morbidities. (**B**,**D**) Increase in medical costs according to the number of morbidities.

Figure 3 represents a key finding of the present study, demonstrating that the cumulative burden of major neonatal morbidities was associated with incremental increases in NICU LOS and medical costs. Similar stepwise patterns were observed across gestational age and birth weight strata, indicating that cumulative morbidity burden adds clinically meaningful burden beyond baseline maturity-related risk. The cumulative number of morbidities was significantly associated with NICU LOS. In the overall population, each additional morbidity was associated with an increase in LOS (β = 8.0 d, 95% confidence interval (CI) 7.9–8.2, *p* < 0.001). When stratified by gestational age, this association remained significant across all groups, with the strongest effect observed in infants born at <25 weeks (β = 8.4 d), followed by those at 32–36 weeks (β = 3.8 d) and 25–31 weeks (β = 2.9 d) (all *p* < 0.001) (Figure 3A). When stratified by birth weight, this association remained significant across all groups, with the strongest effect observed in infants weighing < 500 g (β = 7.28 d, 95% CI 5.2–9.4, *p* < 0.001), followed by those weighing 1500–2500 g (β = 4.97 d, 95% CI 4.8–5.2, *p* < 0.001), 500–999 g (β = 4.01 d, 95% CI 3.2–4.8, *p* < 0.001), and 1000–1499 g (β = 3.17 d, 95% CI 2.7–3.6, *p* < 0.001) (Figure 3A).

A similar graded association was observed for medical costs. In the overall population, each additional morbidity was associated with a significant increase in medical costs (β = 32,526,000 KRW, 95% CI 31,889,000–33,162,000, *p* < 0.001). Stratified analyses demonstrated that this association persisted across all gestational age groups, with the largest incremental increase observed in infants born at 32–36 weeks, followed by those at 25–31 weeks and <25 weeks (all *p* < 0.001) (Figure 3B). Analyses also showed that this association persisted across all birth weight groups, with the largest incremental increase observed in infants weighing 1500–2500 g (β = 43,346,000 KRW), followed by those weighing 1000–1499 g (β = 22,200,000 KRW), <500 g (β = 18,131,000 KRW), and 500–999 g (β = 13,748,000 KRW) (all *p* < 0.001) (Figure 3B).

In both panels, outcomes increased linearly with the number of morbidities across all gestational age groups. The incremental increase in LOS was most pronounced in infants born at <25 weeks, whereas the increase in medical costs was steeper in infants born at 32–36 weeks. Values represent the estimated increase per additional morbidity derived from regression models.

Overall, the cumulative burden of neonatal morbidities demonstrated a clear stepwise increase in relation to both NICU LOS and medical costs. Meanwhile, temporal analyses indicated relatively stable overall trends despite heterogeneous variability across individual morbidities.

### 3.4. Impact of Individual Neonatal Morbidities on Length of Stay and Medical Costs

**Figure 4:**
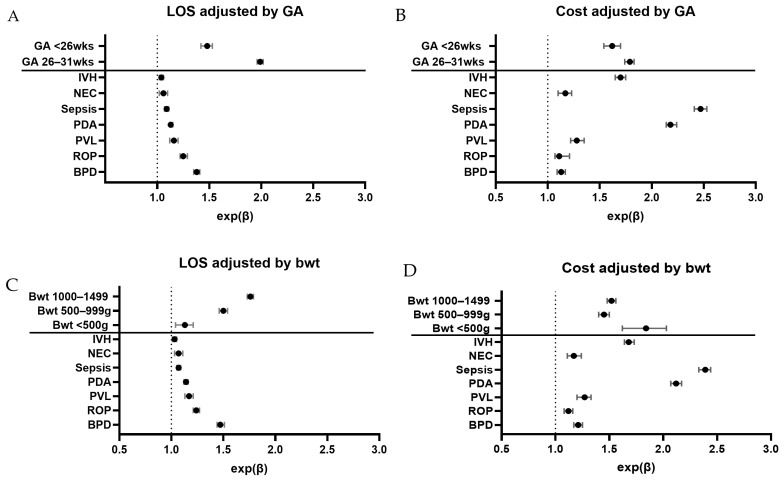
Forest plots of multivariable generalized linear model (GENMOD) analyses for NICU LOS and medical costs adjusted for gestational age and birth weight. All major neonatal morbidities were independently associated with increased LOS and medical costs after adjustment for gestational age (GA) and birth weight (bwt). Data are presented as exp(β) with 95% confidence intervals, representing the multiplicative relative increase in LOS or medical costs associated with each morbidity. Reference groups were infants without the corresponding morbidity (=1), GA 32–36 weeks (**A**,**B**), and birth weight 1500–2500 g (**C**,**D**). BPD, bronchopulmonary dysplasia; bwt, body weight; GA, gestational age; IVH, intraventricular hemorrhage; LOS, length of stay; NEC, necrotizing enterocolitis; PDA, patent ductus arteriosus; PVL, periventricular leukomalacia; ROP, retinopathy of prematurity.

After adjustment for gestational age and birth weight, all major neonatal morbidities were associated with increased NICU LOS and medical costs (Figure 4).

Gestational age and birth weight showed stronger associations with NICU LOS than any individual neonatal morbidity. Compared with infants born at 32–36 weeks of gestation, those born at <26 weeks and 26–31 weeks had substantially prolonged LOS after adjustment, with increases of approximately 13–21 days. Similarly, lower birth weight groups showed progressively longer LOS compared with infants weighing 1500–2500 g at birth.

Among neonatal morbidities, BPD showed the strongest independent association with prolonged LOS after adjustment for gestational age or birth weight. In the identity-link model, BPD was associated with approximately 13–15 additional hospital days, followed by ROP (+7 days) and PDA or PVL (+3–4 days). Corresponding exp(β) estimates from the log-link model indicated that BPD was associated with approximately 1.5-fold longer LOS, whereas ROP and PDA were associated with approximately 20–30% longer LOS relative to infants without these morbidities. IVH, NEC, and sepsis showed relatively smaller effect sizes after multivariable adjustment.

For medical costs, gestational age and birth weight also demonstrated strong associations. However, morbidity-related effect sizes appeared relatively more pronounced for medical costs than for LOS. PDA and sepsis showed the strongest associations with increased medical costs, followed by IVH, PVL, NEC, BPD and ROP.

Overall, gestational age and birth weight were the primary determinants of NICU LOS, whereas specific neonatal morbidities, particularly BPD, PDA, and sepsis, contributed additional independent burdens on hospitalization duration and healthcare expenditures.

## 4. Discussion

In this nationwide cohort study, we demonstrated that both individual neonatal morbidities and their cumulative burden are strongly associated with prolonged NICU LOS and increased medical costs. While gestational age and birth weight remain fundamental determinants of NICU LOS, our findings highlight the cumulative impact of multiple coexisting morbidities on both clinical and economic outcomes. These results suggest that gestational age and birth weight establish the baseline for hospitalization, whereas the superimposed burden of morbidities serves as the strongly associated variability in both LOS and medical costs among preterm infants.

A key finding of this study is the clear stepwise escalation of both NICU LOS and medical costs according to the cumulative number of major neonatal morbidities. This finding, presented in Figure 3, highlights that morbidity burden is an important determinant of healthcare utilization across all strata of prematurity. Each additional morbidity was associated with an approximately 8 d increase in LOS, a trend that remained remarkably consistent even after adjustment for gestational age and birth weight. Notably, the slope of this increase was more pronounced in the most immature infants (gestational age < 25 weeks), suggesting that each additional complication imposes a greater burden due to the infant’s underlying physiological fragility. This cumulative effect underscores the importance of integrated clinical strategies aimed at preventing multiple complications rather than managing individual conditions in isolation. Previous studies have reported that infants admitted to or transferred to tertiary-level hospitals tend to have longer NICU LOS, likely reflecting higher clinical severity and the need for more complex care [19].

Our data highlight a notable discrepancy between NICU LOS and economic expenditure. In particular, the financial impact of cumulative morbidities was even more striking than the increase in LOS. Each additional diagnosis was associated with a significant incremental rise in total hospitalization costs, driven not only by the prolonged bed occupancy but also by the intensive use of high-cost resources such as specialized medications and procedures, surgery, nutritional support, and diagnostic imaging required for complex cases. Interestingly, Figure 3C,D suggest that the incremental increase in medical costs associated with each additional morbidity is proportionally more pronounced in relatively more mature preterm infants. Although extremely preterm and extremely low birth weight infants have higher baseline LOS and medical costs, additional morbidities in relatively mature infants may represent a greater deviation from the expected clinical course. This finding suggests that neonatal morbidities impose a substantial economic burden across the full spectrum of prematurity, not only among the most immature infants.

In addition to the baseline effects of prematurity, BPD showed the most profound association with prolonged LOS—likely due to the protracted need for weaning from respiratory support and delayed discharge readiness characteristic of chronic pulmonary morbidity. In contrast, sepsis and PDA showed stronger associations with medical costs despite relatively smaller effects on LOS. This suggests that the economic burden in the NICU is driven not only by bed days but also by high-intensity interventions, expensive pharmacotherapy (such as surfactants or specialized antibiotics), and frequent diagnostic imaging and multidisciplinary care associated with acute complications. Importantly, surfactant replacement therapy in Korea is generally administered according to standardized national reimbursement criteria, including neonates with respiratory distress syndrome requiring mechanical ventilation with fractional inspired oxygen (FiO_2_) > 0.3 and characteristic clinical/radiographic findings, as well as prophylactic administration for high-risk preterm infants with a birth weight ≤ 1250 g or gestational age < 30 weeks. Because the HIRA database is based on reimbursed insurance claims, surfactant therapy included in this study were likely limited to those meeting these standardized reimbursement indications, thereby reducing variability related to discretionary overuse or defensive medical practice. These findings emphasize that prioritizing neonatal interventions requires a dual perspective: targeting BPD to reduce hospital occupancy and system-level strain, while concurrently optimizing the management of acute infections and PDA to mitigate direct healthcare expenditures.

At a systems level, the observed variability in NICU LOS suggests that discharge readiness is not a purely biological milestone but is influenced by institutional practice patterns and reimbursement frameworks. In the context of Korea’s national health insurance system, structured reimbursement policies—ranging from per diem components to specialized procedure fees—may create distinct incentives compared to diagnosis-related group-based systems. Previous studies have reported that infants covered by public insurance tend to have longer lengths of hospitalization [20], suggesting that reimbursement structures may influence clinical decision-making and discharge practices. In Korea, where NICU care is supported by a national health insurance system, such structural factors should be considered when interpreting LOS and resource utilization. A recent nationwide quasi-experimental study evaluating the expansion of public insurance coverage for NICU admission in Korea reported no significant improvement in infant or under-five year age mortality, despite increased access to intensive care [21]. These findings suggest that healthcare system factors, including reimbursement policies, may influence resource utilization without necessarily improving outcomes. This perspective is consistent with our findings that increased morbidity burden is associated with greater healthcare utilization, highlighting the complex relationship between clinical severity, system structure, and resource use.

Finally, the economic implications extend beyond the hospital ledger to the family unit. The strong association between cumulative morbidity and cost underscores the potential for financial toxicity among families of medically complex infants. Given that parental anxiety regarding future healthcare costs is often high yet under-addressed in clinical settings, our study provides a quantitative basis for clinicians to engage in earlier, more transparent financial counseling. Preparing families for the long-term economic trajectory is an essential component of comprehensive family-centered care in the NICU.

This study has several strengths. First, its nationwide cohort enhances generalizability across diverse clinical settings. Second, the large sample size enables robust evaluation of both individual morbidities and their cumulative effects. Third, the integration of clinical outcomes with cost data provides a comprehensive assessment of the burden of neonatal complications. Unlike single-center studies, our analysis also captures variations in practice patterns and patient populations across Korea, highlighting the contribution of system-level factors to NICU LOS.

Some limitations should be acknowledged. First, as an observational analysis, causal relationships cannot be established. Second, detailed clinical decision-making processes or social factors influencing discharge may not have been fully captured. Because neonatal morbidities were identified using administrative claims codes rather than detailed clinical chart review, misclassification bias may have occurred. In addition, detailed procedure-level information, including the number of invasive catheterizations, catheter-days, and peripherally inserted central catheter or umbilical catheter use, was not available in the HIRA claims database. Therefore, the potential contribution of invasive procedures to medical costs, sepsis risk, and prolonged NICU LOS could not be directly evaluated. Third, variations in institutional discharge criteria may have influenced the observed differences in LOS. Although we partially accounted for inter-institutional heterogeneity by applying generalized linear mixed models with hospital as a random effect, information regarding hospital type (e.g., public versus private institutions), referral patterns, and hospital-level practice variation was not available in the database and therefore could not be fully adjusted for in the analysis. These unmeasured institutional factors may have influenced resource utilization and LOS across NICUs. Also, due to the limitations of retrospective studies, causal inferences were restricted, even though the findings suggested strong associations between these factors and increased length of hospitalization and costs. A final limitation of this study is that infants who died during hospitalization were included in the analysis. This may have introduced a competing-risk bias, as infants with the highest clinical severity may experience shorter LOS because of early mortality. Consequently, the association between severe neonatal morbidities and prolonged hospitalization or increased medical costs may have been underestimated among surviving infants with similar disease burdens.

## 5. Conclusions

Our nationwide population-based study demonstrates a significant correlation between the cumulative burden of neonatal morbidities and both NICU LOS and total medical costs. We found that as the number of co-existing morbidity increases, the healthcare resource utilization escalates disproportionately. These findings underscore the importance of integrated clinical management to mitigate multiple morbidities, which is essential not only for improving neonatal outcomes but also for reducing the long-term socioeconomic burden. Our results provide critical baseline data for establishing more efficient healthcare resource allocation and insurance policies for the high-risk neonatal population in Korea.

## Data Availability

The data used in this study were obtained from the Korean Health Insurance Review and Assessment (HIRA) database. Restrictions apply to the availability of these data, which were used under license for the current study and are therefore not publicly available. Data may be available from the HIRA upon reasonable request and with permission from the relevant authorities.

## Abbreviations

The following abbreviations are used in this manuscript:

BPD	Bronchopulmonary dysplasia
CI	Confidence interval
GA	Gestational age
GLMM	Generalized linear mixed model
GENMOD	Multivariable generalized linear model
HIRA	Health Insurance Review and Assessment Service
ICD	International Classification of Diseases
IRB	Institutional Review Board
IVH	Intraventricular hemorrhage
KCD	Korean Standard Classification of Diseases
KRW	Korean Won
LOS	Length of stay
NEC	Necrotizing enterocolitis
NICU	Neonatal intensive care unit
PDA	Patent ductus arteriosus
PVL	Periventricular leukomalacia
ROP	Retinopathy of prematurity
SGA	Small for gestational age
USD	United States dollar

## Appendix A

### Operational Definitions and ICD-10 Diagnostic Codes

**Table A1:** Operational definitions and ICD-10 diagnostic codes used to identify major neonatal morbidities.

Morbidity	Operational Definition	ICD-10 Codes
Bronchopulmonary dysplasia (BPD)	Presence of BPD diagnosis during NICU hospitalization	P27.1
Patent ductus arteriosus (PDA)	Presence of PDA diagnosis during hospitalization	Q25.0
Neonatal sepsis	Presence of neonatal sepsis diagnosis during hospitalization	P36.x
Intraventricular hemorrhage (IVH)	Presence of IVH diagnosis during hospitalization	P52.x
Retinopathy of prematurity (ROP)	Presence of ROP diagnosis during hospitalization	H35.1
Necrotizing enterocolitis (NEC)	Presence of NEC diagnosis during hospitalization	P77
Periventricular leukomalacia (PVL)	Presence of PVL diagnosis during hospitalization	P91.2

**Table A2:** ICD-10 diagnostic codes used to specify birth weight of under 1500 g.

ICD-10 Codes	Term
P070.0	Birth weight, less than 500 g
P070.1	Birth weight, 500–749 g
P070.2	Birth weight, 750–999 g
P070.9	Extremely low birth weight, unspecified weight
P071.0	Birth weight, 1000–1249 g
P071.1	Birth weight, 1250–1499 g

**Table A3:** ICD-10 diagnostic codes used to identify prematurity.

ICD-10 Code	Term
P07.20	Extreme immaturity of newborn, unspecified weeks of gestation
P07.21	Extreme immaturity of newborn, gestational age less than 23 completed weeks
P07.22	Extreme immaturity of newborn, gestational age 23 completed weeks
P07.23	Extreme immaturity of newborn, gestational age 24 completed weeks
P07.24	Extreme immaturity of newborn, gestational age 25 completed weeks
P07.25	Extreme immaturity of newborn, gestational age 26 completed weeks
P07.26	Extreme immaturity of newborn, gestational age 27 completed weeks
P07.30	Preterm newborn, unspecified weeks of gestation
P07.31	Preterm newborn, gestational age 28 completed weeks
P07.32	Preterm newborn, gestational age 29 completed weeks
P07.33	Preterm newborn, gestational age 30 completed weeks
P07.34	Preterm newborn, gestational age 31 completed weeks
P07.35	Preterm newborn, gestational age 32 completed weeks
P07.36	Preterm newborn, gestational age 33 completed weeks
P07.37	Preterm newborn, gestational age 34 completed weeks
P07.38	Preterm newborn, gestational age 35 completed weeks
P07.39	Preterm newborn, gestational age 36 completed weeks
